# Powder metallurgy inspired low-temperature fabrication of high-performance stereocomplexed polylactide products with good optical transparency

**DOI:** 10.1038/srep20260

**Published:** 2016-02-03

**Authors:** Dongyu Bai, Huili Liu, Hongwei Bai, Qin Zhang, Qiang Fu

**Affiliations:** 1 College of Polymer Science and Engineering, State Key Laboratory of Polymer Materials Engineering, Sichuan University, Chengdu 610065, P. R. China

## Abstract

Stereocomplexation between enantiomeric poly(l-lactide) (PLLA) and poly(d-lactide) (PDLA) provides an avenue to greatly enhance performance of eco-friendly polylactide (PLA). Unfortunately, although the manufacturing of semicrystalline polymers generally involves melt processing, it is still hugely challenging to create high-performance stereocomplexed polylactide (sc-PLA) products from melt-processed high-molecular-weight PLLA/PDLA blends due to the weak crystallization memory effect of stereocomplex (sc) crystallites after complete melting as well as the substantial degradation of PLA chains at elevated melt-processing temperatures of ca. 240–260 °C. Inspired by the concept of powder metallurgy, here we report a new facile route to address these obstacles by sintering of sc-PLA powder at temperatures as low as 180–210 °C, which is distinctly different from traditional sintering of polymer powders performed at temperatures far exceeding their melting temperatures. The enantiomeric PLA chain segments from adjacent powder particles can interdiffuse across particle interfaces and co-crystallize into new sc crystallites capable of tightly welding the interfaces during the low-temperature sintering process, and thus highly transparent sc-PLA products with outstanding heat resistance, mechanical strength, and hydrolytic stability have been successfully fabricated for the first time.

With the growing awareness on sustainability issues associated with traditional petroleum-derived and nonbiodegradable polymers, developing biodegradable polymers derived from renewable resources (e.g., corn starch) has aroused considerable attention in recent years^1^,^2^. As one of such promising eco-friendly polymers, polylactide (PLA) exhibits a tremendous application potential in various fields from biomedical engineering to recyclable industrial packaging materials owing to its excellent biocompatibility, outstanding transparency, impressive mechanical strength and stiffness, easy processability, and competitive price^3^,^4^,^5^. Unfortunately, although most physical properties are equivalent to those of generally used engineering plastics such as polystyrene (PS) and poly(butylene terephthalate) (PBT), the current incorporation of PLA into large-scale commercial applications has been greatly restricted by several obstacles, especially its inferior heat resistance limited by the relatively low melting temperature (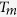) of 150–180 °C and poor long-lasting durability related to the hydrolytic degradation during its service life^6^,^7^,^8^. Even for one major application as textile fibers, both the heat resistance and the hydrolytic stability of PLA is not high enough to avoid deformation and hydrolysis after dyeing at fairly low temperature of about 110 °C^9^. Very interestingly, the past two decades have witnessed the formation of stereocomplex (sc) crystallites by blending of equimolar poly(l-lactide) (PLLA) and poly(d-lactide) (PDLA) as one effective strategy to significantly improve these properties of PLA^10^,^11^,^12^,^13^. Because the two PLA chains with opposite configurations align alternatingly and pack tightly in the crystal lattice through strong intermolecular hydrogen-bonding interactions, pure sc crystallites display an extremely high 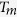 of around 50 °C higher than that of PLLA or PDLA homochiral (hc) crystallites^14^,^15^,^16^ and subsequently provides a possibility to give PLA-based materials a much better dimensional stability at high temperatures (the highest heat distortion temperature can reach to 200 °C^10,11^). Moreover, it has been proved that the stereocomplexed polylactide (sc-PLA) shows many superior properties such as much better mechanical strength^10^,^11^,^12^ and hydrolysis resistance^10^,^13^.

The physicochemical properties of sc-PLA are strongly dependent on the level of sc crystallinity and the molecular weight of PLA enantiomers^10^,^11^,^12^,^13^,^17^. High sc content and PLA molecular weight (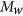 ≥ 10^5^) generally result in high performances of sc-PLA, particularly regarding the mechanical strength, heat resistance, and hydrolysis resistance. Nevertheless, the exclusive formation of high-content sc crystallites cannot easily be realized either from the melt of high-molecular-weight (HMW) PLLA/PDLA (50/50) blends or from their solution since stereocomplexation is competed with the formation of hc crystallites^18^,^19^,^20^,^21^,^22^. On the other hand, sc crystallites formed in the HMW PLLA/PDLA blends have a poor melt memory effect to restore sc crystallization after complete melting^23^,^24^,^25^,^26^,^27^. When pure sc crystallites are melted and crystallized again, both sc crystallization and hc crystallization take place simultaneously and competitively. Therefore, the industrially meaningful melt-processing technology commonly used to fabricate polymer products with desired forms and shapes, such as injection molding, allow only the formation of a HMW sc-PLA product containing a mixture of both hc and sc crystallites. More importantly, despite the immense interests for developing effective methods (e.g., introducing stereoblock PLA^25^, adding nucleating agent^26^,^27^, and solid-state cross-linking^17^) to improve the melt stability of sc crystallites, conventional melt-processing of HMW sc-PLA at temperatures (>240 °C) typically greater than its 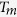 is undesirable because this process inevitably causes substantial degradation of PLA chains^28^. To date, it is still an extremely challenging task to achieve high-performance sc-PLA products using solvent-free processing technology as a facile and effective approach.

Powder metallurgy (PM) is a shaping process of creating near-net metallic parts from compacted metal powders by welding the particle interfaces at temperatures below the 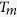 of the powder^29^. It provides an attractive way to magically tackle the dilemma regarding the processing of metallic and alloy materials that are impossible to melt or form in other ways. Inspired from the PM, herein, we report a new and facile strategy for fabricating high-performance sc-PLA products with good optical transparency through low-temperature (as low as 180–210 °C) sintering of powdery sc crystallites for the first time. Because the sintering temperature is just located in the appropriate temperature window (below the 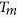 of sc crystallites but well above that of hc crystallites) for sc crystallization^21^,^30^,^31^,^32^,^33^, the enantiomeric PLA chain segments from adjacent powder particles is expected to interdiffuse across the particle interfaces and co-crystallize into new sc crystallites capable of binding the isolated particles into a solid piece. The impressive optical transparency, hydrolytic stability, and thermomechanical properties of these sintered sc-PLA products are highlighted by comparing with those of the compression-molded sc-PLA ones. Furthermore, it is worth mentioning that this low-temperature sintering processing is distinctly different from the commonly used sintering of nascent polymer powder, such as ultra-high molecular weight polyethylene (UHMWPE)^34^,^35^, where typically requires a high temperature above the 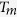 of the powder to make it melted completely.

## Results

**Figure 1:**
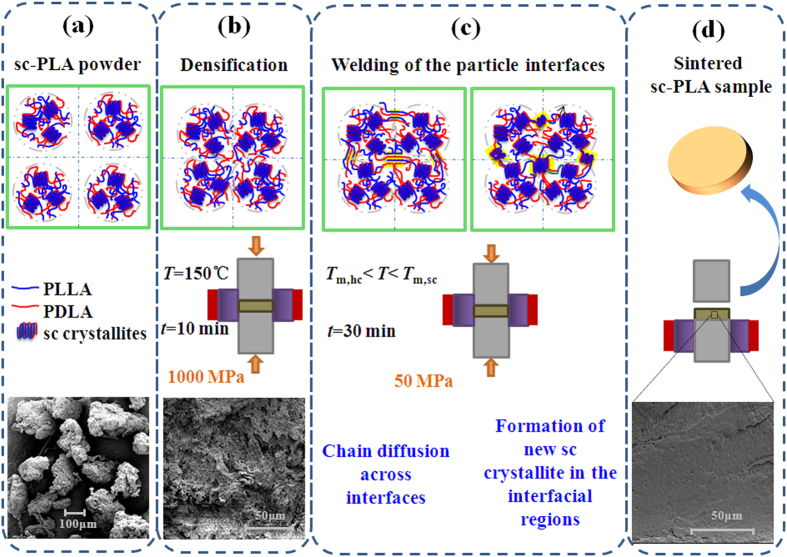
Schematic illustration showing the low-temperature fabrication protocol of sc-PLA products by means of powder metallurgy inspired sintering.

The HMW sc-PLA powder was prepared by solution blending of equimolar PLLA and PDLA, and then the low-temperature sintering of the as-prepared sc-PLA powder was carried out in a standard hydraulic press equipped with a self-made piston-cylinder high-pressure apparatus. As shown schematically in Fig. 1, the sintering protocol used for fabricating sc-PLA products involves two main stages: densification of the sc-PLA powder (around 50–150 μm in size) at 150 °C under a pressure of 1000 MPa to facilitate the wetting of the powder particles (Fig. 1a,b), and then the welding of the isolated powder particles provided by interdiffusion as well as co-crystallization of enantiomeric PLA chain segments across the interfaces of adjacent particles at temperatures of 180–210 °C under a pressure of 50 MPa (Fig. 1c,d).

**Figure 2:**
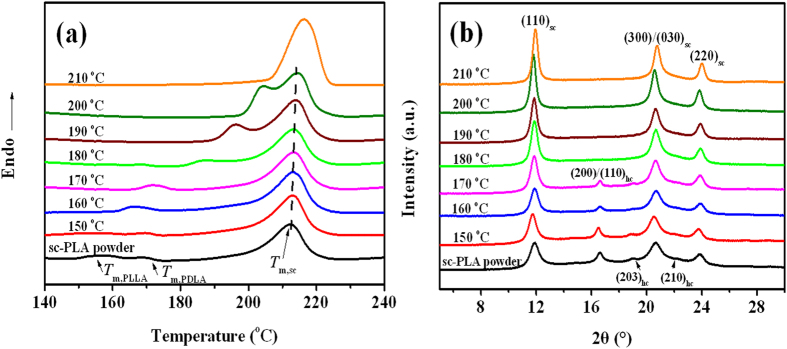
(**a**) DSC melting curves and (**b**) WAXD profiles of sc-PLA powder and its products sintered at different temperatures.

**Figure 3:**
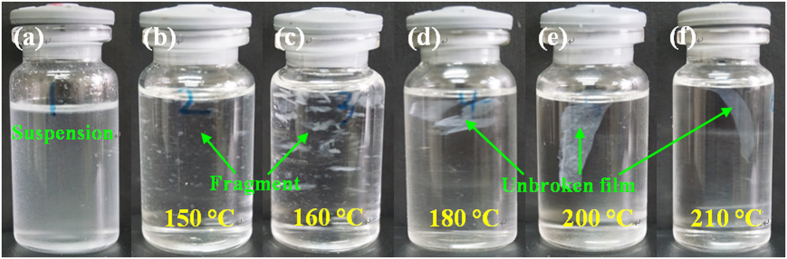
Digital photos of (**a**) nascent sc-PLA powder and (**b–f**) sintered sc-PLA films after dipping in chloroform (5 mg/mL) for two weeks. The sintering temperatures are given in the profile.

In order to provide a clear evidence for the formation of new sc crystallites at the particle interfaces during the low-temperature sintering process, both differential scanning calorimeter (DSC) and wide-angle X-ray diffraction (WAXD) measurements were performed. The DSC heating curves and WAXD profiles of nascent powder and several sc-PLA products sintered at different temperatures are shown in Fig. 2. Obviously, most of the samples display a typical DSC multiple melting behavior (Fig. 2a). The melting peaks in the temperature ranges between 160 and 175 °C is related to the melting of hc crystallites^36^, whereas those at higher temperatures of around 180–215 °C is assigned to the melting of sc crystallites^37^,^38^. For nascent sc-PLA powder, one strong melting peak (at around 215 °C) of sc crystallites along with two small melting peaks of hc crystallites (the one at around 164 °C for PLLA and the other one at 172 °C for PDLA^17^) are observed in the DSC thermogram, indicating the formation of high-purity sc-PLA powder with trace amounts of hc crystallites. It is interesting to find that, after sintering at 180–210 °C, the melting peaks of hc crystallites disappear and another shoulder peak presents before the dominant melting of sc crystallites. The WAXD profiles of these sintered products exhibit three characteristic diffraction peaks of sc crystallites at around 11.9°, 20.7°, 24.3°, corresponding to the (110), (300)/(030), and (220) planes^10^,^39^, but no characteristic peaks of hc crystallites at around 17.0° and 19.0°, attributed to the (200)/(110), (203), and (210) planes^39^,^40^, can be detected in Fig. 2b. Therefore, the appearance of the shoulder peak can be tentatively ascribed to the formation of new sc crystallites composed of thinner lamellae at the particle interfaces. More interestingly, both the magnitude and onset temperature of the shoulder peak increase remarkably with increasing sintering temperature (Fig. 2a), suggesting that the formation of new sc crystallites across the interfaces is strongly dependent on the sintering temperature and relatively high temperature is preferable for the co-crystallization of enantiomeric PLA chain segments into perfect sc crystallites. Also, sintering not only gives rise to a notable enhancement in the sc crystallinity (the fusion enthalpy of 100% crystalline sc crystallites was taken as 142 J/g^10^), from 35.4% for nascent sc-PLA to 53.4% for the sc-PLA product sintered at 210 °C, but also make the primary sc crystallites more perfect as evidenced by the slightly increased melting temperature. With regard to the products sintered at lower temperature of 150–170 °C, the formation of new sc crystallites seems difficult because the chain diffusion could be strongly restricted by the partially-melted hc crystallites (as evidenced by the weakened characteristic peaks of hc crystallites). The formation of new sc crystallites across the particle interfaces of the sintered sc-PLA powders can be further confirmed by a delicately designed dissolution experiment using chloroform as a solvent, as shown in Fig. 3. It is based on the fact that chloroform is a good solvent for hc crystallites while sc crystallites cannot be dissolved in chloroform. As expected, a stable suspension is obtained for nascent sc-PLA powder (Fig. 3a), whereas large pieces of unbroken sc-PLA films are observed for sc-PLA products sintered at temperatures of 180–210 °C because the adjacent sc-PLA powder particles are strongly connected by the newly-formed sc crystallites across the interfaces in these cases (Fig. 3d–f). While for the sc-PLA products sintered at lower temperatures (e.g., 150–170 °C), the sc-PLA powder particles are not connected together as a whole and thus part of the adjacent particles are separated from each other after the uncomplexed PLA chains are dissolved in chloroform (Fig. 3b,c).

**Figure 4:**
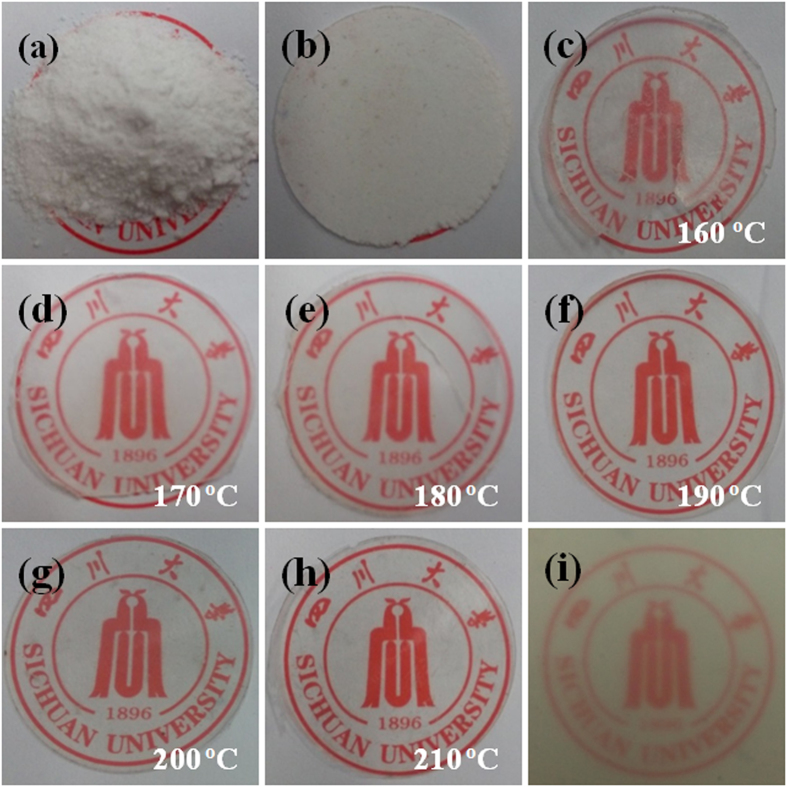
Material appearance of (**a**) nascent sc-PLA powder, (**b**) densified sc-PLA powder, (**c–h**) sc-PLA sheets sintered at different temperatures, and (**i**) sc-PLA sheet prepared by conventional hot pressing at 240 °C. We gratefully acknowledge the permission to use the logo from Sichuan University.

**Figure 5:**
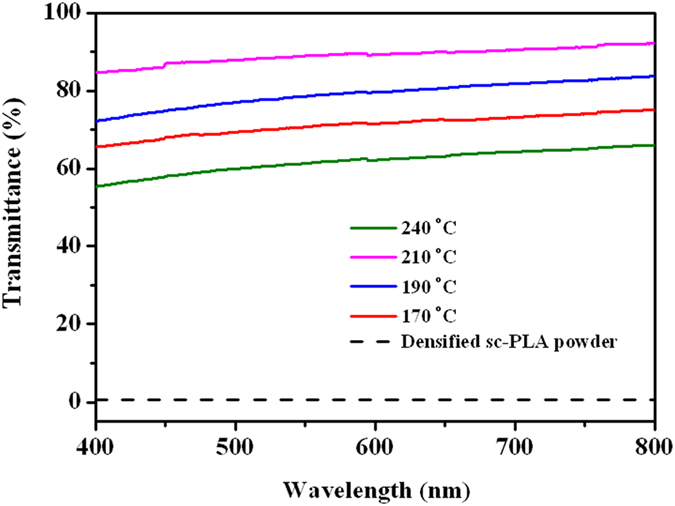
UV-vis transmittance spectra of sc-PLA films sintered at different temperatures and that compression-molded at a temperature of 240 °C. The thickness is ~300 μm for all films.

**Figure 6:**
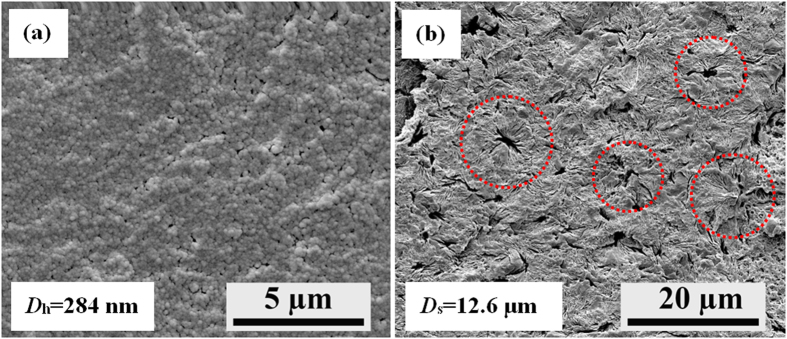
SEM images showing the crystal morphologies of (**a**) sc-PLA sheet sintered at 210 °C and (**b**) that compression-molded at 240 °C.

Figure 4 presents the appearances of the starting sc-PLA powder and some final sheet products. In contrast to the opaque and yellow sc-PLA sheet prepared by conventional compression-molding at 240 °C (Fig. 4i), all the sintered sc-PLA sheets show a much better optical transparency (Fig. 4c–h). Moreover, the transmittance enhances significantly with the increase in the sintering temperature from 170 °C to 210 °C (Fig. 5), distinctly indicating a strong dependence of the optical transparency on the sintering temperature or the welding level of the particle interfaces. Specially, an impressive transparency (the transmittance is above 90% in the visible region) can be obtained in the sc-PLA sheets sintered at 190 °C. Considering that the optical transparency of semicrystalline polymers is strong associated with the crystal size, the crystal morphology of these samples was observed with scanning electron microscope (SEM) and some representative results are presented in Fig. 6. For the compression-molded sc-PLA sheet, the average diameter (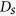) of PLA spherulites (as indicated by the circles in Fig. 6a) is about 12.6 μm, much larger than the wavelength of visible light (390–780 nm). However, the sc crystallites in the sintered sc-PLA sheet are very tiny and thus they cannot be easily distinguished (Fig. 6b). The average size (hydrodynamic diameter, 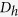) of the SC crystallites determined by dynamic light scattering (DLS) is as low as 284 nm, so the sintered sc-PLA sheets exhibit superior transparency. The reduction in transmittance with the decrease of sintering temperature is ascribed to the scattering effect of the unwelded interfaces between adjacent sc-PLA powder particles.

**Figure 7:**
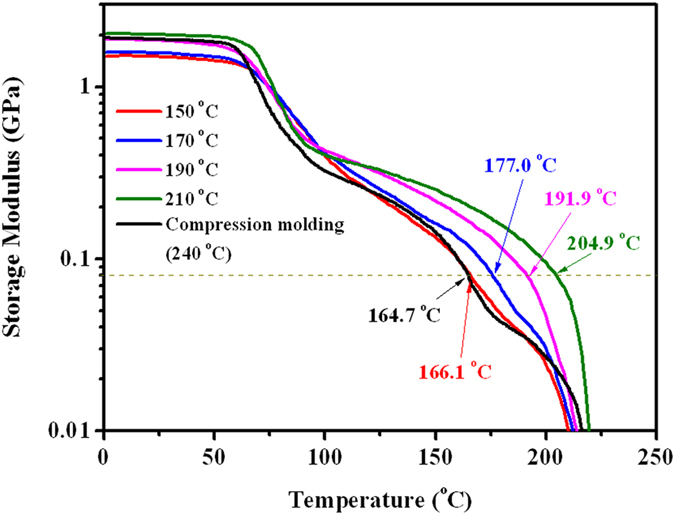
Storage modulus vs. temperature curves of sc-PLA sheets sintered at different temperatures and that compression-molded at a temperature of 240 °C.

To highlight the important role of interfacial welding in determining the performances of the sintered sc-PLA products, the heat resistance of some products was analyzed with dynamic mechanical analysis (DMA). Dynamic storage modulus (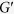) was measured from 0 to 250 °C at a heating rate of 3 °C min^−1^ and the results are given in Fig. 7. Evidently, the sc-PLA product sintered at a low temperature of 150 °C share the same poor heat resistance with the compression molded products at 240 °C. The value of 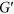 drops considerably when the temperature exceeds 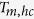 due to the absence of sufficient sc crystallites to effectively strengthen the interfaces between adjacent powder particles. However, with the increase in the sintering temperature up to 210 °C, the heat resistance of the sc-PLA products is improved dramatically. Its 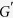 (ca. 80 MPa) at 204.9 °C is even comparable to that for compression-molded product at 164.7 °C, distinctly indicating that high-performance sc-PLA products can only be achieved when the interfaces of the sintered powder are tightly welded by the crystalline network of sc crystallites formed at appropriate sintering temperatures.

**Figure 8:**
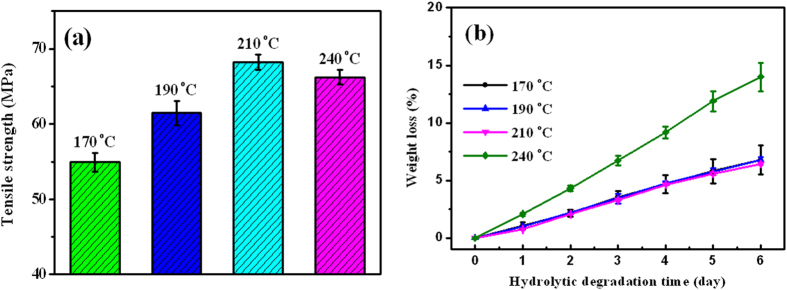
(**a**) Tensile strength and (**b**) hydrolytic degradation rate of sc-PLA sheets sintered at different temperatures and that compression-molded at a temperature of 240 °C.

Figure 8 presents the tensile strength and hydrolytic degradation rate of the as-prepared sc-PLA sheets. Expectedly, the tensile strength of the sintered sc-PLA sheets increases greatly with increasing sintering temperature from 170 °C to 210 °C (Fig. 8a). Moreover, a tensile strength of 68.2 MPa is achieved in the sc-PLA sheet sintered at 210 °C, which is apparently higher than that of the sheet compression-molded at 240 °C. More importantly, it is very interesting to find that all the sintered sc-PLA sheets share almost the same superior hydrolytic stability (Fig. 8b).

In summary, we have demonstrated a simple but robust strategy to fabricate high-performance, optically transparent sc-PLA products by means of low-temperature powder sintering for the first time. The unique characteristic of co-crystallization between PLLA and PDLA chains allows for a significantly reduction of processing temperatures versus conventional melt processing technologies. At temperatures as low as 180–210 °C, just below the melting temperature (215–220 °C) of sc crystallites, the enantiomeric PLA chain segments from adjacent powder particles can not only interdiffuse across particle interfaces but also co-crystallize into new sc crystallites in the interfacial regions to strongly bind the initially isolated particles together. Very interestingly, the interfacial-localized crystalline network can impart the sintered sc-PLA sheet products with outstanding heat resistance, impressive transparency, good mechanical strength, and favorable hydrolytic stability. We believe this work could open a promising way towards industrial-scale fabrication of sc-PLA-based products using conventional polymer sintering equipments.

## Methods

### Materials

PLLA (trade name 4032D) with a D-isomer content of 1.4% and a weight-averaged molecular weight (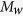) of 1.7 × 10^5^ g·mol^−1^ was obtained from NatureWorks LLC, U.S.A. PDLA with a D-isomer content of 99.5% and a 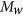 of 1.2 × 10^5^ g·mol^−1^ was supplied by Zhejiang Hisun Biomaterial Co., Ltd, China.

### Preparation of sc-PLA powder

sc-PLA in the form of powder was prepared by solution blending of equimolar PLLA and PDLA. Briefly, equal amounts of PLLA and PDLA were separately dissolved in chloroform (40 g·L^−1^) at room temperature to generate two different transparent solutions. After then, the prepared solutions were mixed together with vigorous stirring, followed by precipitating the formed sc-PLA using excessive methanol and further vacuum-drying at 40 °C for about three days to remove residual solvent.

### Low-temperature fabrication of sc-PLA sheet products

sc-PLA sheets were prepared by compacting the as-prepared sc-PLA powder using a standard hydraulic press (Chengdu Zhengxi Hydraulic Equipment Manufacturing Co., Ltd, China) equipped with a self-made piston-cylinder high-pressure apparatus of tungsten carbide, as sketched in Supplementary Figure 1. The sample powder can be sealed in the sample cell (24 mm in diameter) between the two pistons and the hydrostatic pressure imposed on the sample can be increased to above 2.0 GPa. An electric heating collar controlled by a temperature controller was used to regulate the temperature of the sample. During the experiment, the magnitude of pressure was measured via an accurate pressure meter and the sample temperature was *in situ* monitored via a thermocouple mounted near the sample cell.

The temperature and pressure protocol used for the fabrication of sc-PLA sheet products is described in Supplementary Figure 2. The fabrication process involves two main stages. (I) Densification: a given amount of sc-PLA powder was filled into the sample cell and heated up at 150 °C for 10 min under a pressure of 1000 MPa, which is one of the most suitable densification conditions previously validated by density measurement. (II) Welding of the particle interfaces by chain diffusion and cocrystallization: the densified sample was heated up to a predetermined sintering temperature in the range of 150–210 °C (close or above the melting temperature of hc crystallites (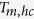) but well below the melting temperature of sc crystallites (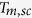)) under a constant pressure of 50 MPa, and kept at this temperature for 30 min to complete the welding process. The sc-PLA product with a diameter of 24 mm and a thickness of 0.5 mm was obtained by cooled down the sintered sample to room temperature at a rate of about 3 °C/min and ejected from the cell after releasing the pressure.

For comparison, conventional compression molding was also used to prepared sc-PLA sheet with a thickness of 0.5 mm on a hot press (KT-0701, China) at 240 °C under a pressure of 10 MPa.

### Characterizations

Scanning electron microscope (SEM) observation was performed using a FEI Inspect F field emission SEM (FE-SEM, U.S.A.) operating at 5 kV. Specimens used for the observation of crystal morphology were prepared by cryo-fracturing the as-prepared samples in liquid nitrogen, followed by etching the amorphous phase away with a methanol-water (2:1 in volume ratio) solution containing 0.025 mol/L of NaOH. Before the SEM observations, the specimen surfaces were sputter-coated with a thin layer of gold.

Thermal analysis was conducted on a Perkin-Elmer pyris-1 differential scanning calorimeter (DSC, U.S.A.) from 50 °C to 250 °C at a heating rate of 10 °C·min^−1^ in a dry nitrogen atmosphere.

Wide-angle X-ray diffraction (WAXD) measurement was performed to characterize the crystalline structure. WAXD patterns were recorded using a Philips X’Pert pro MPD diffractometer with Cu Kα radiation (λ = 0.154 nm) operating at 40 kV and 40 mA. During the measurement, the specimens were scanned from 5 to 40° at a 2θ scanning rate of 5° min^−1^.

Dynamic mechanical analysis (DMA) was conducted on TA Q800 equipment (U.S.A.) in a tensile mode with a strain of 10 μm and a frequency of 1 Hz. Dynamic storage modulus (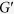) was recorded during heating from room temperature to 250 °C at a scanning rate of 3 °C·min^−1^. Rectangular specimens (20 mm in length and 2 mm in width) used for the dynamic mechanical testing were cut from the sintered disks.

Dissolution experiment was carried out by dipping the specimens in chloroform (5 mg/mL). The digital photos were taken after the mixtures were sealed in glass bottles for two weeks.

Dynamic light scattering (DLS) measurement was performed using a Brookhaven BI-200SM apparatus (U.S.A.) at room temperature. The suspension of the sc-PLA powder for light scattering were prepared with chloroform (0.5 mg/mL) and filtered through a 0.45 μm filter prior to use. The scattering angle and He-Ne laser wavelength were set as 90° and 633 nm, respectively. The detectable particle size range was 2 nm to 1 μm.

Tensile properties were tested on dumbbell-shaped tensile bars using a SANS universal tester (China) at a cross-head speed of 5.0 mm/min. The geometry of the parallel section of the tensile bars is 15 × 4 × 1 mm. All the tests were performed at room temperature and the average value was obtained from at least six independent bars for each sample.

Hydrolytic degradation of the specimens (10 mm × 10 mm × 1 mm) were performed in NaOH solution (pH = 13) at 37 °C. After a predetermined period of time (i.e., 24 h), the specimens were removed from the NaOH solution, washed thrice with distilled water, and vacuum dried to get a constant weight. The degradation rate was evaluated by the variation of weight loss with degradation time. For each sample, the average value of the weight loss was derived from at least five specimens.

Optical transmittance of the specimens with a thickness of 300 μm was measured using an INESA L5 UV-vis spectrophotometer (China) in the wavelength range from 400 to 800 nm.

## Additional Information

**How to cite this article**: Bai, D. *et al*. Powder metallurgy inspired low-temperature fabrication of high-performance stereocomplexed polylactide products with good optical transparency. *Sci. Rep*. **6**, 20260; doi: 10.1038/srep20260 (2016).

## Supplementary Material

Supplementary Information
