# Supplementary material for: Powder metallurgy inspired low-temperature fabrication of high-performance stereocomplexed polylactide products with good optical transparency
Source: Sci Rep. 2016 Feb 3;6:20260. doi: 10.1038/srep20260 (PMC4738299; doi:10.1038/srep20260)
Supplement: Supplementary Information [file srep20260-s1.doc]

**Supplementary information**

**Powder metallurgy inspired low-temperature fabrication of high-performance** **stereocomplexed polylactide products with good optical transparency**

Dongyu Bai, Huili Liu, Hongwei Bai^*^, Qin Zhang, Qiang Fu^*^

College of Polymer Science and Engineering, State Key Laboratory of Polymer Materials Engineering, Sichuan University, Chengdu 610065, P. R. China

Corresponding author. Tel./Fax: +86 28 85461795. E-mail: bhw_168@163.com (H.W. Bai), qiangfu@scu.edu.cn (Q. Fu).


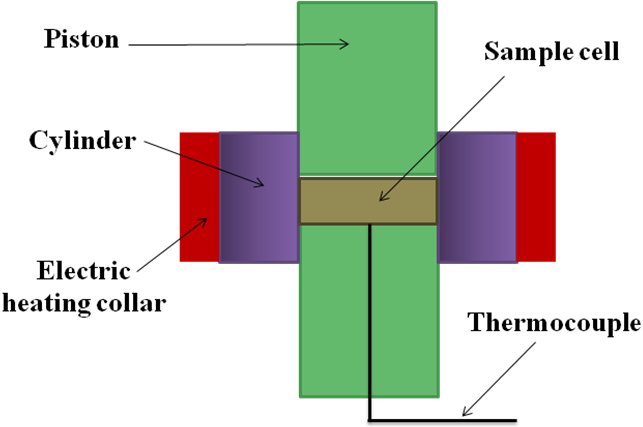


**Supplementary Figure 1.** Schematic illustration of the self-made piston-cylinder high-pressure apparatus.


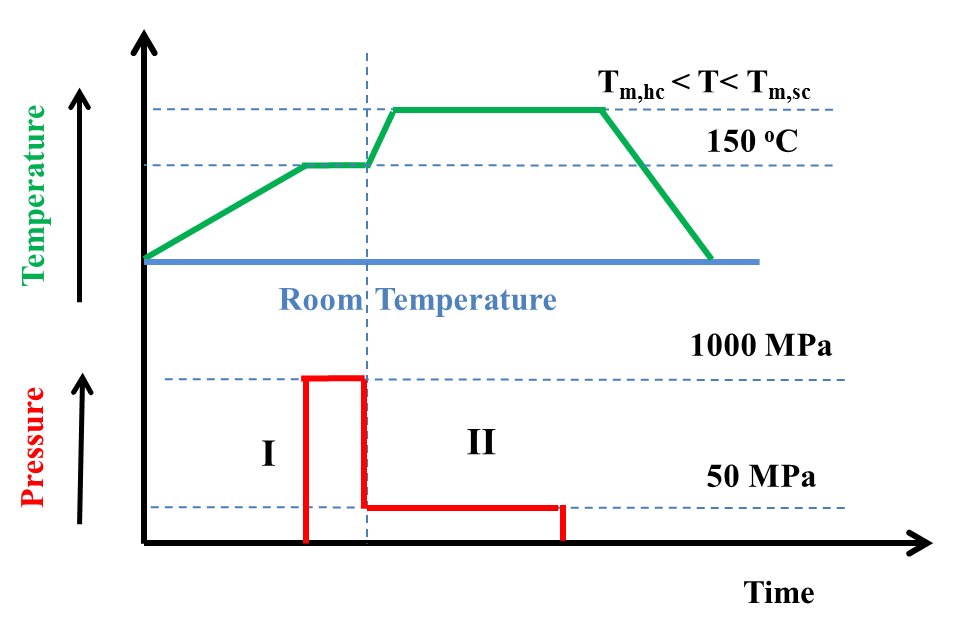


**Supplementary Figure 2.** Sketch of the temperature and pressure protocol used in the low-temperature sintering of sc-PLA powder.
